# An Interplay Between Reaction-Diffusion and Cell-Matrix Adhesion Regulates Multiscale Invasion in Early Breast Carcinomatosis

**DOI:** 10.3389/fphys.2019.00790

**Published:** 2019-08-13

**Authors:** Dharma Pally, Durjay Pramanik, Ramray Bhat

**Affiliations:** Department of Molecular Reproduction, Development and Genetics, Indian Institute of Science, Bangalore, India

**Keywords:** breast cancer, multiscale invasion, cell adhesion, reaction diffusion, cellular potts model (CPM)

## Abstract

The progression of cancer in the breast involves multiple reciprocal interactions between malignantly transformed epithelia, surrounding untransformed but affected stromal cells, and the extracellular matrix (ECM) that is remodeled during the process. A quantitative understanding of the relative contribution of such interactions to phenotypes associated with cancer cells can be arrived at through the construction of increasingly complex experimental and computational models. Herein, we introduce a multiscale three-dimensional (3D) organo- and pathotypic experimental assay that approximates, to an unprecedented extent, the histopathological complexity of a tumor disseminating into its surrounding stromal milieu via both bulk and solitary motility dynamics. End point and time-lapse microscopic observations of this assay allow us to study the earliest steps of cancer invasion as well as the dynamical interactions between the epithelial and stromal compartments. We then simulate our experimental observations using the modeling environment Compucell3D that is based on the Glazier–Graner–Hogeweg model. The computational model, which comprises adhesion between cancer cells and the matrices, cell proliferation and apoptosis, and matrix remodeling through reaction–diffusion–based morphogen dynamics, is first trained to phenocopy controls run with the experimental model, wherein one or the other matrices have been removed. The trained computational model successfully predicts phenotypes of the experimental counterparts that are subjected to pharmacological treatments (inhibition of N-linked glycosylation and matrix metalloproteinase activity) and scaffold modulation (alteration of collagen density). Further parametric exploration-based simulations suggest that specific permissive regimes of cell–cell and cell–matrix adhesions, operating in the context of a reaction–diffusion–regulated ECM dynamics, promote multiscale invasion of breast cancer cells and determine the extent to which the latter migrate through their surrounding stroma.

## Introduction

**Figure 1:**
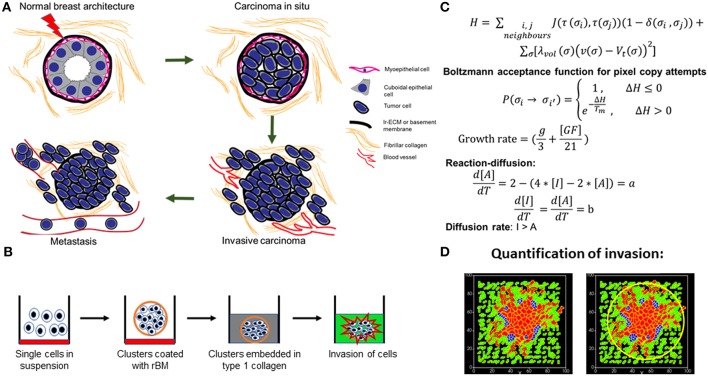
Schematic depiction of experimental system and computational model. **(A)** Early stages of breast cancer progression: Top left denotes the normal glandular (ductal/luminal) architecture of human breast. Top right denotes the pattern of breast epithelia undergoing ductal carcinoma *in situ* where normal epithelia are malignantly transformed, lose polarity, and proliferate, resulting in filling up of ductal lumen. Bottom right shows the architecture of invasive ductal/luminal carcinoma where transformed cells breach the basement membrane (BM) and invade into collagen-rich breast stroma. Bottom left shows how invasive breast cancer cells, having traversed through stroma intravasate into blood/lymph vessels and metastasize to secondary organs. **(B)** Schematic workflow of 3D invasion assay used throughout the paper: Cells are first cultured on top of nonadhesive substrata in medium containing 4% reconstituted BM (rBM). Once cells assembled into clusters, the latter is embedded within type 1 collagen scaffolds and cultured in serum-free conditions. **(C)** Governing equations used for setting up the computational model. The first equation calculates *H*, which is Hamiltonian of the simulation: *H* determines the probability *P* associated with index-copy attempts, movements by generalized cells to minimize local effective energy through the default dynamical algorithm known as modified Metropolis dynamics. The equation for calculating growth rate shows it to be dependent on [GF], the growth factor concentration, and *g*, which denotes nutrient availability. Extracellular matrix (ECM) remodeling follows the kinetics of reaction-diffusion (please refer to the appropriate sections in Material and Methods for a more detailed description of model construction). **(D)** Quantification of invasiveness of cancer cells in the computational model is performed using the minimal enclosing circle algorithm developed using MATLAB.

Within physiologically functioning tissues and organs, cells constantly interact with their surrounding extracellular matrix (ECM). This complex and continual interaction is essential for organ development and homeostasis (Bhat and Bissell, 2014; Bhat and Pally, 2017). Alterations that affect cell–ECM interactions aid in the progression of pathologies like cancer (Nelson and Bissell, 2005; Simi et al., 2018). In normal mammary glands and breasts, luminal epithelial cells are surrounded by a layer of myoepithelial cells that secrete basement membrane (BM): a sheet-like ECM rich in laminin and non-fibrillar collagens. Such mammary epithelial architectures are surrounded by stromal ECM that is rich in fibrillar matrix proteins such as collagen I (Figure 1A) and connective tissue cells such as fibroblasts, macrophages, and adipocytes. In breast cancer, this architecture is lost: the lumen is filled with proliferating apolar transformed epithelia, myoepithelia are absent, and the BM is ultimately breached by invading cells (Polyak and Kalluri, 2010). The stroma shows degradation of ECM, fibrosis, leucocytic infiltration, neoangiogenesis, and lymphangiogenesis (Wiseman and Werb, 2002; Orimo et al., 2005; Dumont et al., 2013). Malignant transformation results in a recalibration of existent interactions with novel constituents and interactions comprising the tumor microenvironment. Studying and quantifying the contribution of a given interaction to the progression phenotype of cancer spatiotemporally are a challenge, as our histo- and biochemical analyses are limited to distinct stages of breast cancer from various patients. Three-dimensional (3D) organotypic and pathotypic cultures of cancer cell lines and primary patient cells have helped extend our understanding of the molecular mechanisms underlying cancer (Torras et al., 2018; Weinhart et al., 2019). The 3D cultures are approximations of the histopathological complexity of *in vivo* tumor microenvironments. Current models involve embedding cancer epithelia within natural or tunable synthetic matrix scaffolds (Balachander et al., 2015; Bhat et al., 2016; Furuta et al., 2018). More complicated versions comprise efforts to mimic the perivascular and endothelial metastatic niches (Ghajar et al., 2013; Carlson et al., 2019) as well as efforts to engineer platforms consisting of multiple organs-on-a-chip reviewed by Zhao et al. (2019).

Noncancerous and malignant breast cell lines, when cultured in reconstituted BM (rBM) matrix, cluster into discrete morphologies that have been described as “round,” “mass,” “grape,” and “stellate” in increasing order of aggressiveness (Kenny et al., 2007). The round phenotype is characteristic of untransformed cells that form growth-arrested acinar-like multicellular clusters with basoapical polarity, and a lumen. Mass and grape phenotypes are characteristic of malignantly transformed epithelia which mimic carcinoma *in situ* or indolently progressive cancers with cells that have completely lost their polarity. The stellate phenotype is typical of highly metastatic cancer cells that actively migrate, although in a collective manner, into and through rBM matrices. Using such 3D assays of cells embedded in rBM, it is possible to study the role of specific expressed proteins in regulating the adhesion between cancer cells or with ECM proteins such as laminins. However, such culture frameworks are inadequate for investigations into the spatial dynamics of cellular transitions between two matrix microenvironments that have distinct rheological properties, such as the non-fibrillar BM-like microenvironment and its fibrillar collagen-like types (Figures S1A,B shows scanning electron micrographs of nonfibrillar rBM and fibrillar type 1 collagen matrices; see Supplementary File 1 for legends of all Supplementary Figures). In addition, it is infeasible to design experiments to observe the phenotypic consequences of an exhaustive exploration of interaction space within a multicomponent biological system.

The second limitation can especially be mitigated by adopting a computational approach and simulating the progression of cancer-like phenotypes for a diverse range of interactive parameter combinations. Computational models, particularly the Cellular Potts model (CPM), have been shown to be useful for such efforts (Zhang et al., 2011; Swat et al., 2012). For example, the deployment of proteolytic and non-proteolytic mode of cancer migration through collagenous scaffolds, or between solitary and collective cell invasion, has been well elucidated using *in silico* approaches (Kumar et al., 2016). However, to the best of our knowledge, no theoretical model has explicitly explored the transitioning dynamics, and the consequences thereof, of cancer cells moving between dissimilar matrix microenvironments. Moreover, while the dynamical role of individual mesoscale physicochemical processes has been well studied in cancer and development (Grant et al., 2004; Zhang et al., 2011; Pantziarka, 2016), whether their combined deployment constrains or widens the phenotypic reaction norm, the spectrum of discrete and distinct phenotypes achievable by cancer cells has not been investigated.

In this paper, we present a unified experimental–computational framework to investigate the interactions between cancer epithelia and spatially compartmentalized ECM microenvironments. The experimental model allows us to break down the phenomenon of cancer cell migration into cellular interactions with the BM, their remodeling of the same, their transition from BM to type 1 collagen, and the subsequent remodeling of, and migration within, type 1 collagen. We closely train the computational model on experimental results. The computational model successfully predicts results of the cancer epithelia upon pharmacological perturbations or scaffold modification. The trained theoretical model also predicts that emergent interplay between reaction–diffusion (R-D) and cell–matrix adhesion can explain the diversity in the extent and mode of invasion of breast cancer cells.

## Materials and Methods

### Cell Culture

MDA-MB-231 cells were maintained in DMEM:F12 (1:1) (HiMedia, AT140) supplemented with 10% fetal bovine serum (Gibco, 10270). MCF-7 cells were grown in Dulbecco's Modified Eagle Medium (DMEM) (HiMedia, AT007F) supplemented with 10% fetal bovine serum. Immortalized Human Mammary Epithelial Cells (HMLE) cells were a gift from Dr. Robert Weinberg, Harvard Medical School, and Dr. Annapoorni Rangarajan, Indian Institute of Science, and were cultured in DMEM:F12 (1:1) supplemented with 1% fetal bovine serum, 0.5 μg/ml hydrocortisone (Sigma, H0888), 10 μg/ml insulin (Sigma, I6634), and 10 ng/ml recombinant human epidermal growth factor (hEGF) (HiMedia, TC228). All the cells were cultured in a 37°C humidified incubator with 5% carbon dioxide.

### Preparation of Cancer Cell Clusters

Normal/cancer cells were trypsinized using 1:5 diluted 0.25% trypsin and 0.02% EDTA (HiMedia, TCL007). A total of 30,000 cells per 200 μl of defined medium (Blaschke et al., 1994) supplemented with 4% rBM (Corning, 354230) were cultured on 3% polyHEMA (Sigma, P3932)-coated 96-well plate for 48 h in a 37°C humidified incubator with 5% carbon dioxide.

### 3D Invasion Assay

rBM-coated clusters were collected into 1.5-ml tubes, centrifuged briefly, and then the supernatant is removed (Figure 1B). Acid-extracted rat tail collagen (Gibco, A1048301) was neutralized on ice in the presence of 10X DMEM with 0.1 N NaOH such that the final concentration of the collagen is 1 mg/ml. Pellet of clusters was resuspended in 50 μl of neutralized collagen and seeded in eight-well chambered cover glass (Eppendorf 0030742036) and supplemented with defined medium. 3D cultures were grown in a 37°C humidified incubator with 5% carbon dioxide.

### Processing of 3D Invasive Clusters

3D invasion cultures were washed with phosphate-buffered saline (PBS) (pH 7.4) once after removing medium and fixed with 4% neutral buffered formaldehyde (Merck, 1.94989.0521) for 30 min. Glycine (2%) in PBS was used to neutralize traces of formaldehyde and was blocked for 1 h at room temperature with 5% bovine serum albumin (BSA) (HiMedia, MB083) in PBS +0.1% Triton X-100 (HiMedia, MB031). After blocking, clusters were stained with 4′,6-diamidino-2-phenylindole (DAPI) (Invitrogen, D1306) and Alexa Flour 633-conjugated phalloidin (Invitrogen, A22284) overnight at 4°C. The next day, cultures were washed with PBS +0.1% Triton X-100 for 10 min each three times.

### Laser Scanning Confocal Microscopy and Time-Lapse Imaging

Processed clusters were imaged using laser scanning confocal microscope (Zeiss LSM 880) with system-optimized Z intervals. Images were analyzed using the Zen Lite software. Brightfield time-lapse imaging was done using Olympus IX81 equipped with stage top incubator and 5% carbon dioxide (see Video 1). Imaging was done at 10 min interval over 24 h. Images acquired were analyzed using the ImageJ software (Schindelin et al., 2012).

### Computational Model

#### Modeling Environment

A multiscale modeling environment is required to simulate the spatiotemporal dynamics within a biological milieu wherein cellular growth, proliferation, invasion and morphogenesis occur simultaneously. The software package CompuCell3D (Swat et al., 2012) fulfills this purpose. Compucell3D is based on the CPM, also known as the Glazier–Graner–Hogeweg model, which was designed to model the collective behavior of active matter (Sanyal and Glazier, 2006; Chen et al., 2007). This is done by calculating an energy function called Hamiltonian at each step of the simulation. Each *cell* (we italicize this term to distinguish CC3D *cells* from biological cells in this paper) is represented by the set of all lattice sites or pixels sharing same cell ID. A rectangular lattice has been used in all our simulations. The evolution of the model happens at each Monte Carlo step (MCS), which consists of index copy attempts of each pixel in the cell lattice. Output of each MCS depends on the Hamiltonian calculation denoted by H (Figure 1C). The Hamiltonian in our model has two contributors which are affected by different properties sum of the *cells* and chemicals. The first contributor is the sum over all neighboring pairs of lattice sites *i* and *j* with associated contact energies (*J*) between the pair of cells indexed at those *i* and *j*. In this term, *i, j* denotes index of pixel, σ denotes *cell* index or ID, and τ denotes cell type. The δ function in this term will ensure that only the σ_*i*_ ≠ σ_*j*_ terms are calculated and also contact energies are symmetric. The contact energy between two *cells* can be considered to be inversely proportional to adhesion between those two *cells*. The second contributor is a function of the volume constraint on the *cell*, where for cell σ, λ_vol_(σ) denotes the *inverse compressibility* of the cell, ν(σ) is the number of pixels in the cell (*volume*), and *V*_t_(σ) is the *cell's target volume*. For each *cell*, this term is governed by its growth equation. If any change in the Hamiltonian is negative at a given MCS for any configuration with respect to its previous one [Δ*H* = (*H*_f_ – *H*_i_) < 0], then index copy attempts of pixels resulting in that configuration will be successful. Otherwise, the attempt will be accepted with probability *P* = exp (−Δ*H*/*T*_m_). A default dynamical algorithm known as modified Metropolis dynamics with Boltzmann acceptance function is used at each MCS to move the system toward a low-energy configuration as MCS increases. The term *T*_m_ can be considered as temperature or magnitude of effective membrane fluctuations. In the model, the membrane fluctuation is kept high for cancer cells compared with matrix elements in order to strike a distinction between living and dead elements. Random movements of pixels leading to different transition probabilities at each MCS mimic the stochasticity present in biological systems. We have modeled the movement of cells in metastasis as guided by differential adhesion and R-D-regulated degradation of ECM surrounding the cells. The MCS can be considered to be the natural unit of time in the model. In biological contexts, MCS and experimental time are considered to be proportional with respect to each other (Alber et al., 2004; Cickovski et al., 2007; Swat et al., 2012).

#### Model Components

##### Cell and matrix orientation

Using a 2D computational model, several aspects of cancer invasiveness and tumor-associated 3D phenomena have been studied where the property of spherical symmetry of tumor morphologies was used to obtain the minimalistic setup (Jiao and Torquato, 2011). Our 2D simulations mimic experiments in which biological cells may require 3D space to allow certain interactions, but in the computational model, only the properties associated with *cells* will play a role in determining the output irrespective of 2D or 3D. 2D simulations are computationally more efficient as it carries out an exponentially smaller number of calculations for the whole system. Our model space is 100 × 100 × 1 pixel in size where a group of cancer *cells* is initially located at the center grid surrounded by ECM. Any element of the model that is required to actively participate through MCS pixel copy attempts must be assigned a *cell* type, as instructed, the laminin (“BM”) and type 1 collagen (“C1”) are assigned different *cell* types along with cancer cells (“CELL”). In the setup, clusters of cancer *cells* are surrounded by blob-like two-*cell* layer of BM. The BM, in turn, is surrounded by fibrillar collagen. To mimic *in vivo* ECM architecture, BM is modeled as dense adhesive blob-like “*cells*” similar to the lamina densa of basal lamina, whereas C1 is modeled as the interconnected fibers similar to type 1 collagen. All components of the system have a depth of 1 pixel in the *z* direction, so there is no overlapping of objects. A *cell* cannot cross another *cell* if it does not degrade it, and without degradation, the *cell* will be trapped in a zone due to steric hindrance by its surrounding environment or find ways to squeeze through small spaces in its vicinity, which become accessible by random movement of that *cell*. In an initial configuration, cancer *cells* start as a rectangular objects of 16-unit volume (4 × 4 pixels) spanning 14 × 14 × 1 unit volume at the center (*x, y* = 43:57) of the simulation grid without any intercellular space. Tightly packed BM *cells* of 9 unit volume (3 × 3 pixels) is then created around the cancer mass (*x, y* = 37:63) having two layers of laminin *cells* separating it from C1. C1 is formulated around the cancer and BM structure throughout the whole grid with initial configuration of 4 unit volume cells (2 × 2 × 1 pixels) with two pixel gaps in between them. In order to make the C1 fibrillar, a plugin is applied on the *cells* which elongate them in the axis, random with respect to each other at 0.8-unit volume increment at each MCS until 5. The length scale of the components of ECM (BM and C1) is kept relatively smaller than the cancer cells (Das et al., 2017). The lattices with no assigned *cell* type or, in other words, the gaps or the free spaces are assigned *cell* type “medium” as a default of the Compucell3D syntax.

##### Contact energies (differential adhesion)

Compucell3D requires setting interactions between all *cell* types in terms of contact energies. Higher contact energy values between two *cell* types signifies lower adhesion or higher mechanical hindrance between them. This is denoted by the term *J* in the Hamiltonian (*H*). The contact energies are set in our simulation by qualitatively considering interactions between pairs of components of the experimental setup in terms of adhesion or repulsion. After running simulations with a range of values of each contact energy, from all the resultant combinations, an appropriate set of contact energies is taken. The contact energies that were established for model simulations included CELL–CELL, CELL–BM, CELL–C1, CELL–medium. Values of these contact energies can be found in Table 1. The CELL–medium contact energy can be thought in terms of CELL–CELL adhesion of cancer with proportional correlation. Higher cancer CELL–CELL adhesion will give higher CELL–medium contact energy value. Across simulations, contact energies were established qualitatively motivated by transcriptomic findings including in, but not limited to (Kenny et al., 2007). For example, to mimic the decreased expression of E-cadherin in highly invasive cells, CELL–CELL contact energy was increased and the CELL-medium contact energy reduced.

**Table 1:** Contact energies for simulations of discrete cancer morphologies.

**Morphology-wise contact energies**	**Cell-cell**	**Collagen-cell**	**Laminin-cell**
Carcinoma-*in-situ* phenotype	3	24	20
Apolar clusters	30	24	30
Multiscale invasive	35	24	45

##### MMP–tissue inhibitors of metalloproteinase (TIMP) reaction diffusion system

Auxiliary equations in Compucell 3D are used to model chemical fields. These fields store the concentration information of a certain chemical at every location in the simulation grid. Two chemical fields, *A* and *I*, are created which are governed by partial differential equations (PDEs) based on R-D dynamics of an activator and its inhibitor. These fields are incorporated with the GGH algorithm to allow interaction between other simulation components and the fields. The governing equations for these two fields are:

(1) $$\begin{array}{l} \frac{\partial \left[A\right]}{\partial t}=D_{A}\nabla^{2}\left[A\right]+a-\delta_{a}\left[a\right] \end{array}$$

(2) $$\begin{array}{l} \frac{\partial \left[I\right]}{\partial t}=D_{I}\nabla^{2}\left[I\right]+b-\delta_{I}\left[I\right] \end{array}$$

(3) $$\begin{array}{l} b=a=K-\left(c^{*}\left[I\right]-d^{*}\left[A\right]\right) \end{array}$$

Where, [*A*], [*I*]: concentration values for fields *A* and *I*.

*D*_*A*_, *D*_*I*_: diffusion constants of *A* and *I*

δ_*A*_, δ_*I*_: degradation rates of *A* and *I*

*a, b*: secretion rate of *A* and *I*

*t* ≡ MCS

Default parameterizations, *D*_*A*_ = 0.01, *D*_*I*_ = 0.8, δ_*A*_ = δ_*I*_ = 0.003, *K* = 2.0, *c* = 4.0, *d* = 2.0.

Here, *A* is considered as the activated form of matrix metalloproteinases (MMPs). Its activation (or secretion of the activated form, i.e., “*a*”) is assumed to be dependent on its inhibitors (inversely) and on its own concentration (autocatalysis) in the form of equation 3 (the inhibitor TIMP is known to bind to, and inhibit, the membrane-bound matrix metalloproteinase (MT1-MMP), which in turn activates secretory diffusible metalloproteinases) (Bourboulia and Stetler-Stevenson, 2010; Brew and Nagase, 2010). There are numerous variants of MMPs and TIMPs present in biological tissues (Brew and Nagase, 2010). Their production rates and interdependencies are still not known entirely for cancer cells, so a generalized form of MMP–TIMP interaction (*A*–*I* interaction of the model) is assumed in the light of R-D dynamics (Equations 1 and 2). The diffusion rate of MMP is set in the same range that is used by previous literature (Kumar et al., 2016) [that model has *D* = 1.0 × 10^−9^ cm^2^.s^−1^ = 0.025 pixel^2^ s^−1^, as 1 mm = 500 pixels]. The difference in diffusion rates between the models (0.01 instead of 0.025) is due to different scaling of MCS with respect to time (s). All other parameters have been set based on previous literature and by optimization of the model. The diffusion rate of *I* is set higher than *A* to generate localized “activator” field and delocalized “inhibitor” field of the R-D system.

As all proteins have a lifetime, the degradation rate or decay constant associated with *A* and *I* in the model limits the spread of fields. The decay constant is assumed to be similar for *A* and *I* due to paucity of rigorous experimental analyses. In the model, the *A* and *I* fields are secreted at the boundaries of all “CELL”s, which come in contact with ECM. To initialize the *A*–*I* axis, random value of “*a*” (range is from 0 to 4) is assigned to each *cell*, which is in contact with ECM at MCS < 5. The CC3D package has a forward Euler method-based PDE solver, which was used to solve the PDEs (Swat et al., 2012).

##### Matrix degradation and regeneration

Investigations into cancer invasion focus primarily on the degradation of matrix by migrating cells that secrete high levels of MMPs. However, cancer cells, while degrading matrices, also secrete their own matrix, which is known as the cancer matrisome. The mechanical properties of the cancer matrisome, the forces it exerts on cells, and its chemical composition are under intense investigation (Naba et al., 2014, 2016; Socovich and Naba, 2018). Degradation of matrix is assumed to be dependent on the ratio of [*A*] over [*I*]. For each MCS, ECM *cells* access the concentration values of the *A* and *I* fields at each of its pixels, and depending on the ratio, those pixels are either degraded or remain unchanged. This degradation is threshold based. Only pixels with the ratio [*A*]/[*I*] > 2.0 will be converted to the “lysed” form, which is either C_Lysed (C1) or L_Lysed (BM) *cell* type. Motivated by the elegant demonstration by Diambra and colleagues of the regulation of Turing space by cooperativity between the activator–inhibitor reaction dynamics (Diambra et al., 2015), we investigated and observed an appropriate regulatory influence of metalloproteinase degradation dynamics on the relative diffusion between MMP and TIMP (see Supplementary File 2). The degraded matrix is assigned different *cell* types by assuming different properties of the degraded form of BM and C1 as the nondegraded BM and C1 also have different properties. As a cost of degradation, [*A*] and [*I*] are reduced at a maximum of 1.5 unit/MCS in pixels belonging to the “lysed” *cell* types. Matrix regeneration is incorporated into the model by conversion of C_Lysed and L_Lysed into C1 (given that cancer cells secrete predominantly fibrillar collagen-rich matrices) after 20 MCS from the degradation event associated with that “lysed” pixel (Socovich and Naba, 2018; Yuzhalin et al., 2018). The regeneration of matrix is essential to eliminate unnecessary free spaces formed as an artifact of matrix degradation, which takes the computational model closer to its experimental counterpart. All the “lysed” *cell* types are subjected to 0.1 volume decrease at each MCS to mimic dissipation of degraded matrix materials *in vivo*.

##### Cellular growth and proliferation

Growth rate of “CELL” is assumed be a linear combination of nutrient availability at cell boundaries and degradation of matrix. The growth equation is given by,

$$\begin{array}{l} \frac{dV}{dt}=g\;*\;p+\left[GF\right]*\;q \end{array}$$

Where *V* = volume of “CELL”

*g* = measure of nutrient availability

[*GF*] = concentration of GF at center of mass of “CELL”

*p, q* = constants.

Two quantities, the common surface area of a *cell* with its neighboring *cells* (*k*) and the total *cell* surface area (*s*) is accessed to calculate *g* in this equation as *g* = *(s-k)/40*. The denominator in the calculation of *g* is due to the 2D nature of the simulation as a *cell* can be surrounded by other *cells* only in the *xy* plane and not in the *z* axis. The scaling of that extra *cell* surface area without any neighboring *cells* in the *z* axis is provided by the denominator. Another contributor of the growth function is *[GF]*, which mimics the ECM degradation dependence of growth and proliferation (Olivares et al., 2017). The “lysed” *cell* types are programmed to secrete GF at each of its pixel location where the diffusion constant is kept low (0.02) to localize this growth signal to areas of matrix degradation. *p* (= 1/3) and *q* (= 1/21) constant values are set according to the assumed weightage of the two variables in growth equation.

Cell division is incorporated into the cancer cells by a CC3D steppable called “MitosisSteppable” with base function “MitosisSteppableBase.” If any “CELL” reaches a threshold volume of 30 units, then those *cells* will be divided in random orientation. The resultant two *cells* will have volumes half of its predecessor with all other properties kept same as the parent *cell*. In this model, growth rate is directly correlated to proliferation as it determines the volume of the *cell* to reach threshold for cell division.

##### Quantification: invasion of morphology

The quantification for the spread or invasiveness of morphologies has been done in MATLAB using minimal enclosing circle algorithm (Figure 1D). Screenshots captured at different MCS from a simulation are used to track invasion of that model. A program was written where for a screenshot, the image is binarized with respect to “CELL,” which is represented by red color. From that binarized image, centroids of all cells are accessed by the function “regionprops.” In order to find the smallest possible enclosing circle, two bits of information are required, which are position of center and radius of a circle which will encompass all the centroid positions. An arbitrary center for the circle can be selected from which distances are measured to all the centroids. In the smallest possible enclosing circle, center-to-centroid distance will be maximum for the furthest centroid that it needs to cover, and this distance will be the radius of the circle. The function “fminsearch” was used with input of assumed centers and radii (maximum of center-to-centroid distance), which yields a center with minimized radius. Circle formed with this center and radius from “fminsearch” will enclose all the centroids and will be the smallest possible circle to do so. All simulations have “CELL” at the center of the grid as the initial configuration, so the center of smallest enclosing circle can be assumed at the center of that grid to start with, which is also the center of screenshot; this optimizes the programme. Running this program will yield the smallest possible enclosing circle for screenshots at each specified MCS, and the area of this circle is considered as the measure of invasiveness of that phenotype (Figure 1D). In our studies, we have explored whether assigning a microenvironment-autonomous motility to *cells* enhances their invasiveness. To address this, we assigned a random motility direction for the “CELL” *cell* type of the model. All of these *cells* are assigned a different direction and a value of the force acting on its center of mass. This assignment of force is randomized but follows a uniform distribution. In Compucell3D, this active motility is incorporated by using “ExternalPotential” plugin and “cellMotility” steppable. The additional contributing term for calculating change in the Hamiltonian (Δ*H*) is:

$$\begin{array}{l} \Delta H_{AM}=-\;{\vec{F}}_{\sigma \left(i\right)}.{\vec{r}}_{ij} \end{array}$$

where for at any MCS step, for a pixel copy attempt from *i* to *j* of *cell* σ(*i*), the force vector is ${\vec{F}}_{\sigma \left(i\right)}$ and the distance vector between those pixels is ${\vec{r}}_{ij}$. So, their dot product with correct alignment in direction will satisfy the condition to minimize *H* and the *cell* will move along that direction.

We observe that simulating multiscale invasion with active motility does not significantly change invasion compared to the model, where active motility is not implemented (Figure S2).

### Statistics

All the biological experiments were repeated three times independently. All the simulations were repeated at least 10 times and the data are represented as mean ± SEM. Parametric Students' *t*-test was performed with Welch's correction to estimate statistical significance.

## Results

### Breast Cancer Cells Invade From rBM-Like Matrix to Collagen-Rich Matrix Concurrently Across Multiple Spatial Scales

**Figure 2:**
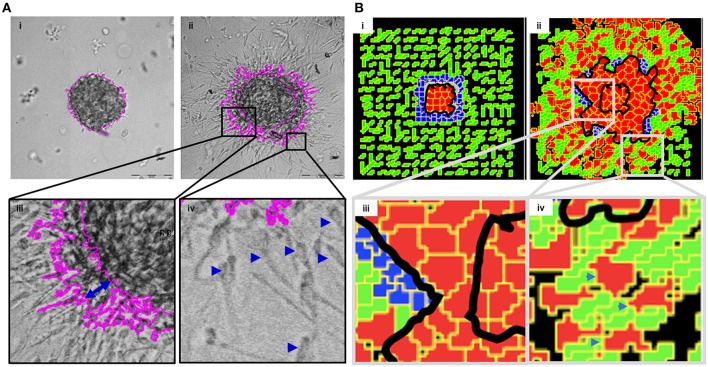
Multiscale multicellular invasion of breast cancer cells in culture. **(A)** Representative phase contrast micrographs from time-lapse imaging of MDA-MB-231 cells showing multiscale invasion into fibrillar matrix. rBM-coated MDA-MB-231 clusters embedded within type 1 collagen **(Ai)** invade into the latter within 24 h **(Aii)**. Cells show expansive migration [**(Aiii)**; double-headed blue arrow shows the extent of collective migration between initial boundary (0 h) and final boundary (24 h) of the cluster (boundaries shown in pink)]. Single mesenchymal cells are also observed in type 1 collagen [**(Aiv)** right inset; blue arrowheads]. Scale bar: 200 μm. **(B)** Multiscale invasion exhibited by computational model. Initial pattern [(**Bi**); MCS10; cancer cells (red) packed within a BM-like nonfibrillar matrix (blue) and further outward by fibrillar collagen-like matrix (green; inter-fibrillar gap = 3 unit pixels)], and final pattern [**(Bii)**; MCS 440] showing invasion of cells. *In silico* cells show expansive migration [**(Biii)** left inset; bulk movement visible through the spatial gap between two black lines denoting boundary at initial MCS and final MCS]. Single cells are also observed in non-fibrillar ECM [**(Biv)** right inset].

In order to mimic the invasion of breast cancer cells *in vivo*, we designed a culture model wherein MDA-MB-231 cells were allowed to form rBM-coated suspended clusters (see Materials and Methods; Figure S3 showing rBM is spatially limited to the surface of clusters). When such clusters were embedded in type 1 collagen (Figure 2Ai) and the cultures were imaged in time lapse, the cancer cells rapidly migrated past the rBM barrier into collagen (Figure 2Aii). We observed spatially distinct but temporally concurrent modes of invasion, ranging from bulk motion, where the cells moved centrifugally in an expansive and collective manner (Figure 2Aiii), to mesenchymal migration of solitary cells with a slender cytoplasmic front and a nucleus-containing lagging end (Figure 2Aiv). We here forward refer to this simultaneous deployment of distinct motility modes as multiscale invasion. Studies concerned with cancer cell migration investigate mechanisms underlying transitions between the modes; however, the studies also note that these modes temporally coexist within histopathological sections of human tumors (Friedl and Alexander, 2011; Friedl et al., 2012; Krakhmal et al., 2015). Our experimental model successfully recapitulates the multimodal and multiscale 3D cancer cell invasion.

We then sought to codify the minimal set of interactive cellular and ECM behaviors that could give rise to such multiscale migratory behaviors of invading cancer cells. Using CC3D, we constructed a computational model wherein for constrained set of values of cell–cell and cell–BM–like ECM adhesion, as well as upon invoking a R-D-based remodeling kinetics of ECM, we observed multiscale invasion of cancer epithelia from a nonfibrillar to a fibrillar *in silico* ECM microenvironment (Figure 2Bi represents the *in silico* cluster at MCS = 10; Figure 2Bii represents the same cluster at MCS = 440; emergence of expansive collective invasion seen in Figure 2Biii, emergence of single cell invasion within the same culture seen in Figure 2Biv; see also appropriate sections in Materials and Methods for details of model construction). The use of an R-D-based modulation of ECM steady state was motivated by the morphology of the invasion phenotypes in our experimental assay, wherein invading cell populations exhibited a discernibly iterative spatial pattern in which invading cells were surrounded by lateral zones of inhibition (activator and inhibitor concentration fields in the simulation shown in Figure S4). In addition, the use of R-D based microenvironmental regulation has strong precedence in the literature on cancer progression (Chaplain, 1995; Gatenby and Gawlinski, 1996; Roque et al., 2018; Zhang et al., 2018). Time series of both bulk and single cell invasion were tracked and found to increase in a similar fashion in culture and in simulations (See Supplementary File 2).

### Nature of the “Stromal” ECM May Determine Mode of Cancer Cell Invasion

**Figure 3:**
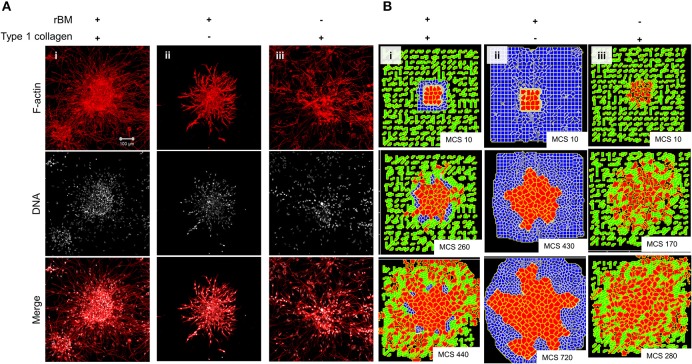
Single-matrix controls of models show simpler modes of invasion. **(A)** Maximum intensity projections of laser confocal micrographs of MDA-MB-231 cell clusters cultured within specific matrix milieu, fixed and stained for F-actin (using phalloidin; red; top row), DNA (using DAPI; white; middle row), and with both signals merged (bottom row). **(i)** rBM-coated clusters embedded in type 1 collagen show multiscale invasion (left column). **(ii)** rBM-coated clusters embedded in rBM show collective or streaming migration of cells, **(iii)** Uncoated MDA-MB-231 clusters in type 1 collagen show predominantly single cell invasion. Scale bar: 100 μm. **(B)** Representative images from simulations of invasion of cancer cells at early (top row), intermediate (middle row), and late MCS steps (bottom row). Simulations mimicking cells encapsulated within nonfibrillar and then fibrillar ECM exhibit multiscale invasion (left column). Simulations of cells cultured exclusively in nonfibrillar and fibrillar ECM show collective and single cell migration (**Bii** and **Biii**). Interfibrillar gap of C1 = 3 unit pixels.

We sought to know whether the multiscale invasion of cancer cells was a function of the prototypical outwardly radial arrangement of cancer cells inside a thin intervening layer of rBM and an outer presence of type 1 collagen. To verify if the initial rBM coating was required for cluster shape and integrity, MDA-MB-231 cells were clustered in the absence of rBM. The cell clusters that formed had an irregular shape with ill-defined contours and were inherently unstable (Figures S5A,B showing irregular and regularly shaped clusters in the absence or presence of rBM coat, respectively). When rBM-coated MDA-MB-231 clusters were cultured entirely in rBM; clusters exhibited collective motility dynamics with most cells still attached to the kernel of the cluster (control multiscale invasion shown in Figure 3Ai; rBM-exclusive control shown in Figure 3Aii). Solitary invading cells were scarcely seen in the periphery. On the contrary, rBM-uncoated clusters upon embedding in type 1 collagen gels rapidly disintegrated into a small kernel and mostly single cells that exhibited mesenchymal single cell migration (Figure 3Aiii).

We used the phenotypic observations to further train our computational model and chose parametric combinations for (i) contact energies of cell–cell, cell–rBM, and cell–type 1 collagen interactions; (ii) R-D-based remodeling of ECM; and (iii) proliferation and death of the cancer cells. We were able to successfully narrow down parametric combinations for which simulations mimicking “only rBM” and “only collagen” controls predicted predominantly collective and single cell migration, respectively (Figure 3Bi represents control, Figure 3Bii shows emergence of collective invasion in an exclusive rBM-like nonfibrillar ECM environment, and Figure 3Biii shows emergence of single cell invasion in an exclusive collagen-like fibrillar ECM environment). Since the parameter combinations were kept identical in the controls, the divergent phenotypes suggest that the identity of the stromal ECM and its spatial arrangement may determine the mode of outward migration of cancer epithelia.

### Metalloproteinase Activity and N-Linked Glycosylation Regulate Multiscale Invasion

**Figure 4:**
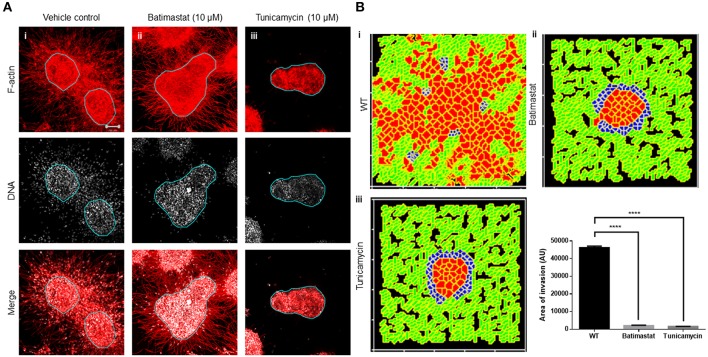
Inhibition of matrix metalloproteinase activity and N-linked glycosylation inhibit multiscale invasion. **(A)** Maximum intensity projections of laser confocal micrographs of MDA-MB-231 cell clusters cultured within specific matrix milieu, fixed and stained for F-actin (using phalloidin; red; top row), DNA (using DAPI; white; middle row), and with both signals merged (bottom row). **(i)** rBM-coated clusters embedded in type 1 collagen treated with vehicle control DMSO show multiscale invasion (left column). **(ii)** Treatment with 10 μM Batimastat leads to inhibition of transition of cells to type 1 collagen although cytoplasmic projections of cells in the periphery of the cluster are visible in the fibrillar matrix. **(iii)** Treatment with 10 μM Tunicamycin results in complete abrogation of multiscale invasion. **(B)** Simulations of control conditions **(i)**, parametric variations analogous with inhibition of R-D **(ii)**, and parametric variations analogous with inhibition of cell–cell, cell–fibrillar ECM, and R-D **(iii)** at MCS590. Graph represents invasiveness of cells in simulations associated with **(Bi–iii)**. Each bar represents mean ± SEM. ^****^p < 0.0001.

We next sought to test our assumption that a locally auto-active regulation of ECM remodeling is essential for multiscale invasion. For MMPs with their cognate lateral inhibitors, TIMPs are putative activator–inhibitor couples, given their diffusivity and the nature of interactions. Treatment of cultures with a broad-spectrum MMP inhibitor Batimastat resulted in an abrogation in transition of cells into the stroma, although the leading cytoplasmic head of cancer cells in the periphery of the cluster could still be visually discerned in the surrounding collagen (Figure 4Ai represents vehicle control; Figure 4Aii represents treatment with 10 μM Batimastat). This suggested that the transition of cancer cell nuclei across the rBM–collagen interface is dependent on protease-dependent remodeling of the stromal ECM. Interestingly, for amoeboid migration (which we have not investigated in our paper, see Discussion), nuclear softening has been proposed to be crucial for protease-independent migration (Das et al., 2019). Decreasing the activator levels within our computational model brought about a decrease in *in silico* migration of cells with sparse transitions into the fibrillar matrix environment (Figure 4Bi represents control; Figure 4Bii represents simulation that shows inhibition of invasion upon downregulating levels of activator A).

Second, we tested the role of cell–cell and cell–matrix interactions by treating our cultures with an inhibitor of N-linked glycosylation: tunicamycin. Tunicamycin affects the glycosylation and trafficking of cell surface proteins (Elbein, 1991). Molecules involved in cell adhesion such as cadherins and cell adhesion molecules (CAMs) are N-glycosylated. Moreover, while E-cadherin expression is epigenetically silenced in invasive MDA-MB-231 cells, N-cadherin is expressed and promotes their motility (Nieman et al., 1999). Treatment with tunicamycin does not alter the trafficking of N-cadherin but affects its function by interfering with its binding to catenin (Youn et al., 2006). Tunicamycin is also known to abrogate the matrix binding functions of integrins (Chammas et al., 1991). The effect of tunicamycin on metalloproteinases is context-dependent (Kim et al., 2010; Lee et al., 2019). Treatment with tunicamycin may increase the expression of MMPs, but due to associated endoplasmic reticulum (ER) stress and unfolded protein response, their secretion is inhibited (Duellman et al., 2015). Treatment of our complex experimental system with tunicamycin completely abrogated stromal transition of cancer epithelia (Figure 4Aiii). The cytoplasmic leading-edge extensions, likely mediated through outside-in integrin signaling, which were observed upon MMP inhibition, were also absent upon tunicamycin exposure.

In our computational model, the phenomenological equivalent of tunicamycin treatment would be to increase contact energies and, hence, downmodulate adhesion between cells and matrices. Additionally, secretion of MMP and TIMP was also downregulated as part of the initial conditions for simulation. Upon doing so, we found impaired invasion of cells into the fibrillar *in silico* environment compared to control conditions (Figure 4Biii). We could also observe inhibition of invasion despite retaining the secretion of MMPs and TIMP but only under parametric combinations when the secretion rate of TIMPs exceeded that of MMPs (Figure S6). Our experimental and computational results suggest that adhesive interactions and local auto-active ECM remodeling dynamics operative within the invading milieu are necessary for stromal migration of cancer cells, and inhibiting them significantly downregulates the latter (Figure 4B).

### Collagen Density Alters Multiscale Invasion

**Figure 5:**
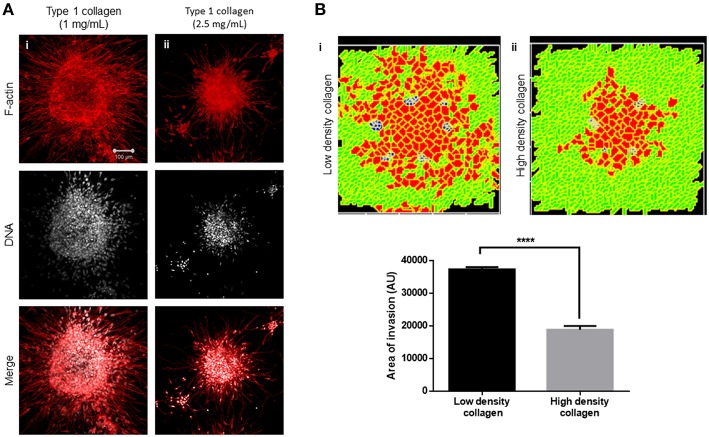
Increased collagen density impairs multiscale invasion. **(A)** Maximum intensity projections of laser confocal micrographs of MDA-MB-231 cell clusters cultured within specific matrix milieu, fixed and stained for F-actin (using phalloidin; red; top row), DNA (using DAPI; white; middle row), and with both signals merged (bottom row). **(i)** rBM-coated clusters embedded in 1 mg/ml type 1 collagen show multiscale invasion. **(ii)** rBM-coated clusters embedded in 2.5 mg/ml type 1 collagen show impaired invasion of cells into surrounding high-density type 1 collagen. **(B)** Simulations of control conditions **(i)** and high-density arrangement of fibrillar ECM showing impaired migration of cells at MCS 540. Graph represents invasiveness of cells in simulations associated with **(Bi,ii)**. Each bar represents mean ± SEM. ^****^p < 0.0001.

We next sought to test whether the arrangement of type 1 collagen fibers surrounding rBM-coated clusters could regulate the nature of cancer cell migration. rBM-coated clusters of MDA-MB-231 cells were embedded within a higher density of type 1 collagen (2.5 mg/ml) scaffolds compared with control (1 mg/ml) (Figure 5Ai). The transition of cancer epithelia into high-density collagen was found to be attenuated (Figure 5Aii). Dense collagen may impede nonproteolytic migration of cancer cells allowing movement only upon mounting a protease-based degradation of ECM. In keeping with our experimental findings, in our computational model, we observe that all other parameters being kept constant, crowding the fibrillar ECM space with a higher density of collagen-like fibers decreased the migration of cells (Figure 5Bi represents control multiscale invasion; Figure 5Bii represents simulation in high-density fibrillar ECM; Figure 5B shows statistically significant impairment of cellular invasion in the computational environment).

### Diversity in Morphological Phenotype Can be Explained by Variation in Interplay Between Cell Adhesion and R-D

Our computational model, trained on controls, successfully predicted the consequences on the phenotype of various perturbations. We asked whether it could also accommodate, with suitable changes in its formalism, the possibility of formation of homeostatic nonmalignant phenotypes as well as precancerous and subinvasive phenotypes? If so, what changes in the underlying coarse-grained physical mechanism could be responsible for those?

**Figure 6:**
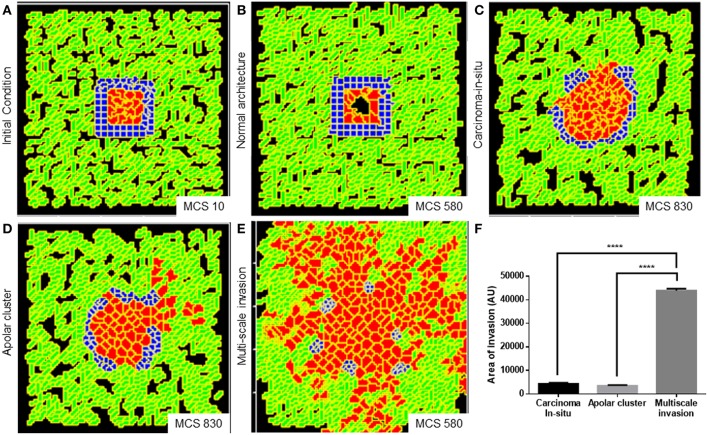
Parameter variation in the computational model can simulate homeostatic, precancerous, and indolently cancerous phenotypes. **(A)** Initial configuration of all components of the computational model at MCS 0. **(B)** Simulation of a homeostatic growth-arrested phenotype with a central lumen obtained upon imposing a nonfibrillar ECM-based rules for regulation of cellular quiescence and death of anchored and detached cells, respectively. **(C)** Simulation of a carcinoma *in situ*-like phenotype obtained by maintaining high values of cell–cell and cell–nonfibrillar ECM adhesion. **(D)** Decreasing cell–cell and cell–ECM adhesion in simulation leads to a phenotype that shows further loss of polarity (as evidenced by a roughness in the outer contour of the clusters) and occasional subinvasive single cell phenotypes. **(E)** Further decreasing cell–cell and cell–matrix adhesion and deployment of an R-D-based kinetics of ECM remodeling leads to multiscale invasion. **(F)** Quantification (bottom right) of the invasiveness of cells from simulations of homeostasis, carcinoma *in situ*, apolar clusters, and multiscale invasion. Scale bar: 100 μm. Each bar represents mean ± SEM. ^****^p < 0.0001.

We obtained a non-invasive homeostatic lumen-containing phenotype (Figure 6A represents phenotype at MCS = 10; Figure 6B represents emergence of the phenotype at MCS = 580) by assigning the cells within our *in silico* framework, certain properties similar to noncancerous ductal epithelial cells: BM-regulated survival of the cells. By simply implementing the rules that (1) cells that are not anchored to the BM-like nonfibrillar ECM die (Frisch and Francis, 1994) and (2) cells anchored to the fibrillar ECM remain quiescent (Spencer et al., 2011), we were able to achieve growth-restricted lumen-containing acini-like structures that resemble the structures formed by the non-malignant cell line HMLE in 3D (Figure S7A). *In silico* phenotypes similar to the pre-cancerous carcinoma *in situ*-like condition, which comprises filled multicellular masses of cells (similar to the mass morphology) (Kenny et al., 2007) (Figure S7B shows MCF7 cells forming similar architectures within our 3D assay) could be observed by increasing intercellular and cell–rBM adhesion (Figure 6C). A more subinvasive morphology, which resembles the precancerous phenotype, but within which cells have lost their polarity and could give rise to indolently progressive tumors, has been referred to as “grape” (Kenny et al., 2007). We simulated outcomes resembling this phenotype upon further relaxing the intercellular and cell–matrix adhesion (Figure 6D). It is crucial to note for simulating both the precancerous and indolent progression phenotypes, the R-D-based ECM remodeling network was not deployed. Invoking the same and decreasing intercellular and cell–rBM adhesion brought about multiscale invasion in simulation (Figure 6E). Comparison of invasiveness between the simulations of three cancerous morphologies (Figure 6F) reveals that multiscale migration exhibits the highest invasiveness followed by the indolently growing cluster phenotype and, in turn, by the precancerous morphological phenotype.

**Figure 7:**
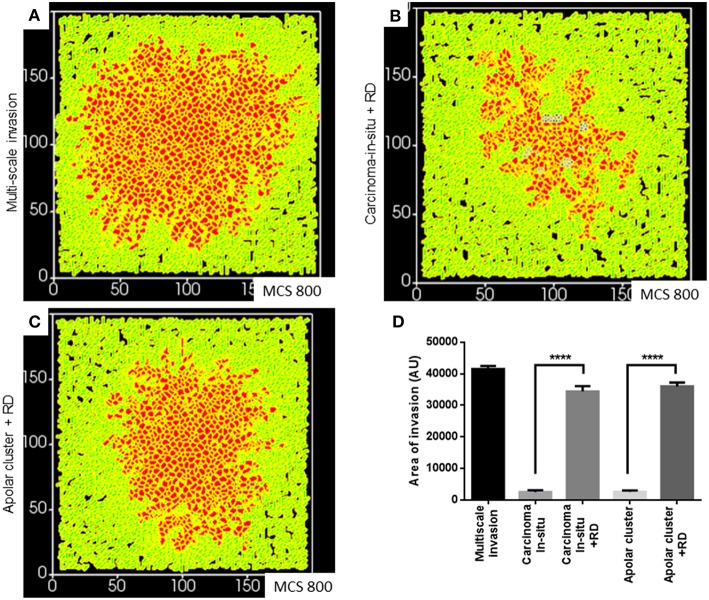
Simulations of the deployment of R-D-based kinetics in carcinoma *in situ* and subinvasive cluster phenotypes. **(A)** Simulation of a control multiscale invasion. **(B)** Simulation, within which regulation of the ECM was modeled using R-D kinetics in parameter regimes of adhesion cognate to carcinoma in situ phenotypes, predicts collective but not single-cell invasion. **(C)** Simulations, within which regulation of the ECM was modeled using R-D kinetics in parameter regimes of adhesion cognate to subinvasive apolar cluster phenotypes, predict multiscale invasion. **(D)** Quantification of the invasiveness of cells from the above simulations in comparison with control multiscale invasion. Each bar represents mean ± SEM. ^****^p < 0.0001.

Finally, we asked whether a decreasing gradient of cell–cell and cell–rBM adhesion was required for increased invasion as predicted by our simulations. Could merely deploying the R-D-based ECM remodeling at higher adhesion regimes bring about greater invasion? Simulating diffusion-driven instability in ECM degradation in the context of the precancerous adhesion parameter values resulted in increased invasion that was exclusively collective and expansive (Figure 7A represents multiscale invasion; Figure 7B represents exclusively collective invasion upon simulating R-D in the context of precancerous adhesion parameter values), and phenocopies the only rBM-like *in silico* morphology (see Figure 3Aii). On the other hand, simulating the same in the context of the adhesion regimes cognate to subinvasive clustered morphologies did result in multiscale invasion (Figure 7C). It is to be noted, however, that the invasion seen in Figures 7B,C was significantly lesser than that of Figure 7A but more than when in such same phenotypes and R-D-based ECM modulation was off (Figure 7D) Our results implicate a threshold that lies between the precancerous and clustered adhesion regimes; the lower the cell and matrix adhesion, the greater the invasion.

## Discussion

In this paper, we adopt a coarse-grained systems–theoretical approach toward the exploration of the mechanisms of stromal invasion of breast cancer epithelia. We designed an experimental organo- and pathotypic culture setup wherein not just the 3D behavior of cancer cells could be studied, but also their transition from non-fibrillar (BM-like) to fibrillar (collagenous) ECM environments, as occurs *in vivo* could also be investigated. Using this assay, we observed epithelial transition both as multicellular collectives and as single mesenchymal cells. In contrast, embedding cells in either (but not both) rBM or collagen (as controls) resulted in predominantly discrete collective and single cell migration, respectively. Our observations imply that the complex multimatrix nature of the assay presented here emulates *in vivo* invasive behavior to a better extent than existent single matrix assays.

Our experimental framework led to the construction, in parallel, of a computational model, whose parameters were trained on the phenotypic outcomes of various experimental controls. The design of the computational model takes inspiration from the concept of dynamical patterning modules (DPMs), autonomous heuristic agents that connote discrete physicochemical phenomena, such as adhesion, differential sorting, R-D, polarity, etc. (Newman and Bhat, 2008, 2009). DPMs, when deployed singly or in combination, are useful for understanding the transformation of cellular patterns in distinct ways. DPMs have been used to investigate mechanisms of developmental morphogenesis in plants and animals (Hernández-Hernández et al., 2012; Niklas and Newman, 2013; Benítez et al., 2018). In addition, a DPM-based understanding of the evolution of development provides an explanation of how body plans of animals showed an accelerated period of origination (known as the Cambrian explosion) followed by a relative stasis (Newman et al., 2009).

In the specific context of breast cancer invasion, using DPMs, we have been able to treat much of the intracellular genetic repertoire and its associated dynamics (mutation and epigenetic regulations) as a black box. Instead, we concentrate on phenotypic traits that manifest at the spatial scales of cells and multicellular populations (Supplementary File 3). We then asked whether specific combinations of parameters pertaining to these traits are permissive to the diversity of morphologies and cellular patterns seen in breast cancer progression. Given discrete assumptions that are confirmed by experiments, the same framework could give rise to phenotypes exhibited by non-malignant, malignant but non-invasive, subinvasive, and aggressively invasive malignant cells. In the case of noncancerous cells, their quiescent and lumen-containing architecture was dependent on adhesion to BM matrix; inability to do so resulted in anoikis (Frisch and Francis, 1994; Schwartz, 1997; Bissell et al., 2002; Furuta et al., 2018). The model predicts that the transition from homeostatic to a precancerous carcinoma *in situ*-like (DCIS) structures involves anchorage-independent survival and division. The transition from DCIS-like states to subinvasive phenotypes that are characterized by complete loss of cell polarity involves a decrease in adhesion, both intercellular and between cells and BM-like matrices. On the other hand, the transition from subinvasive phenotype to a full-blown invasive multiscale phenotype is predicted to be achieved through specific interplay between decreased cell–cell and -matrix adhesion and R-D-based cross-modulation between regulators of ECM remodeling, with neither physical process being sufficient by itself to bring about the phenotype. The computational model upon being asked to deploy R-D in the presence of high cell–cell and cell–matrix adhesion predicted an exclusively collective invasion phenotype. The latter resembles morphologies obtained when cancer cell clusters are cultured in rBM scaffolds in the absence of type 1 collagen. This suggests that the progression between two given morphologies can be achieved through distinct and dissimilar trajectories in parameter space.

R-D-based mechanisms have been proposed to regulate the spatial patterning of iterative structures in development such as hairs, feathers, and digits (Sick et al., 2006; Glimm et al., 2014; Raspopovic et al., 2014). This occurs through interaction between an autocatalytic mediator of a morphogenetic step and its inhibitor (Gierer and Meinhardt, 1972; Meinhardt and Gierer, 2000). Both the mediator and its inhibitor are, as per the R-D formalism, expected to be diffusible in nature (Turing, 1952). Their interaction would lead to spatial foci of morphogenesis separated by lateral zones of inhibition. It is reasonable to hypothesize the mediator to be a negative regulator of a morphological trait and its inhibitor to therefore antagonize the mediator's inhibition of morphogenesis. MMPs and TIMPs are exemplars of such processes. They have been shown to play significant roles in mammary gland branch patterning (Wiseman and Werb, 2002). Their interaction dynamics in the context of mammary morphogenesis and elsewhere has been proposed to act through R-D (Grant et al., 2004; Hoshino et al., 2012; Skaalure et al., 2016; Kumar et al., 2018).

A brief survey of expression patterns of genes across multiple cell lines grown on top of rBM matrices provides support for our predictions (Kenny et al., 2007). Cell lines exhibiting subinvasive and invasive morphologies exhibit a progressive decrease in E-cadherin expression for which experimental support is available (Hiraguri et al., 1998). Cell lines with subinvasive morphologies showed decreased levels of β1 integrin, which participates in multiple integrin heterodimers that bind to laminin. Invasive cells specifically expressed an aberrantly glycosylated levels of a β1 integrin (the consequences of glycosylation of β1 integrin have been reviewed in Bellis, 2004). Invasive cancer epithelia are known to express matrix metalloproteinases to a greater extent than untransformed cells: MDA-MB-231, for example, shows high levels of multiple MMPs as well as TIMP, relative to poorly invasive MCF7 cells (Balduyck et al., 2000; Bachmeier et al., 2001).

The modeling approach we have used successfully distinguishes between collective and single-cell growth dynamics. However, it is not able to parse mesenchymal vs. amoeboid motilities. This is because we have modeled cells as bounded units that show little change in shape as they move. We aim to overcome this limitation in the future by constructing multicompartment cells wherein intracellular cytoskeletal dynamics will be incorporated and will also be allowed to respond to inhomogeneities in ECM patterns. Our black-box approach also assumes a direct intracellular linkage between the various extracellular phenomena that mediate invasion. The introduction of interprocess linkages with added feedbacks, delays, and cooperativities as a means of linking adhesion, proliferation, motility, and ECM remodeling, and the (non)linear dynamics associated with the links would further enrich our understanding of the coordination between the diverse cellular phenomena and will be taken up in future efforts. In our computational model, cells proliferate copiously. On the other hand, our culture assays are grown for 24–36 h; cell proliferation can at best be construed to play a mild role in the overall invasion. These two observations are not inconsistent with each other though; proliferation is also observed in cultures grown for longer time periods but does not alter the pattern of invasion that has been initially set by cell migration. The activator–inhibitor couple in our simulations diffuse through and act on matrices: we have therefore not simulated the effect of boundary constraints on the spatial patterns of cellular invasion as explored by others (Diambra and Costa Lda, 2006). In forthcoming papers, we will supplement the collagenous matrix in our experimental assay with cells such as fibroblasts, macrophages, and noncancerous breast epithelia: therein, we intend to computationally explore the effect of spatial constraints on the R-D-based regulation of MMP-TIMP diffusion.

3D pathotypic cultures from patient cells/organoids are increasingly being considered as standards for personalized therapeutic strategies (Hagemann et al., 2017; Pauli et al., 2017). Their ability to prognose radio- and chemoresistance and match the results of patient-derived xenograft models is backed up by a burgeoning body of literature (Hubert et al., 2016; Zeeberg et al., 2016; Gilles et al., 2018). Most of these culture setups lack a stromal compartment. The addition of the latter, as we have done in our assay, may prove to be a useful spatial milieu wherein the effect of immunotherapeutic interventions is tested. Our experimental breast cancer model can also be adapted for other cancers wherein the effect of stromal constituents on multiscale invasion of transformed epithelia may be studied and targeted.

## Data Availability

The datasets generated for this study are available on request to the corresponding author.

## Author Contributions

DPa and RB designed the experiments. Dpa performed the experiments. DPr and RB designed the simulations. DPr performed the simulations. DPa, DPr, and RB analyzed the results of experiments and simulations and wrote the manuscript.

### Conflict of Interest Statement

The authors declare that the research was conducted in the absence of any commercial or financial relationships that could be construed as a potential conflict of interest.
