# Supplementary material for: An Interplay Between Reaction-Diffusion and Cell-Matrix Adhesion Regulates Multiscale Invasion in Early Breast Carcinomatosis
Source: Front Physiol. 2019 Aug 13;10:790. doi: 10.3389/fphys.2019.00790 (PMC6700745; doi:10.3389/fphys.2019.00790)
Supplement: Supplementary file 2 [file Data_Sheet_1.docx]

Video 1: **Time lapse imaging of invasive cancer cell clusters in type 1 collagen.**

rBM coated MDA-MB-231 cell clusters embedded in type 1 collagen showing multiscale invasion. Scale bar: 200 μm

Figure S1: **Scanning electron micrographs of ECMs used in assays.**

(A) SEM micrograph of rBM shows sheet-like or non-fibrillar architecture. (B) SEM micrograph of polymerized Type 1 collagen shows fibrillar architecture.

Figure S2: **Simulation of multiscale invasion with incorporation of active motility.**

(A) Multiscale invasion of cancer cells when simulated without assigning active motility to the cells (MCS: 540). (B) Simulation showing multiscale invasion of cancer cells when active motility and randomized initial direction was incorporated (MCS 540). (C) Quantification of cancer cells invasion showing no effect of active motility on multiscale invasion (n=5).

Figure S3: **Presence of rBM on the surface of the MDA-MB-231 cell clusters.**

(A) Single optical slice of laser confocal micrograph showing the presence of rBM (stained with pan laminin antibody) exclusively on the surface of the clusters. (B) Maximum intensity projection of the same showing surface staining of pan laminin on MDA-MB-231 cell clusters. (Green: pan-laminin; White: DNA; Red: F-actin) Scale bar = 20 μm.

Figure S4: **Localization of activator (A) and inhibitor (I) during simulation.**

Multiscale invasion of cancer cells during cell field (left column). Heatmap of localization of activator chemical field (A) (middle column) and inhibitor chemical field (I) (right panel) during various steps of simulation (MCS= 20, 420, 650).

Figure S5: **Phase contrast micrographs of MDA-MB-231 cells clusters cultured on non-adherent substrates.**

(A) Formation of rough edged and loosely formed clusters in the absence of rBM after 48 h of culture in suspension (B) Smooth boundary and tightly packed clusters are formed when the cells were cultured in 4% rBM containing medium for 48 h in suspension. Objective:10X

Figure S6: **Simulation of tunicamycin treatment with deployment of reaction-diffusion-based kinetics**

Simulation of treatment of clusters with tunicamycin (A: initial condition) with parametric variations analogous Figure 4B(iii) except in this case, the secretion of TIMP and MMP is not inhibited with the former higher than the latter (B and C at MCS 350 and 590). The graph plotted from statistics of n=10 simulations shows that there is a significant difference between control and in silico treatment with tunicamycin along the lines of experimental data. Each bar represents mean +/- SEM **** denotes p-value <0.0001.​

Figure S7: **Growth arrested, precancerous and multiscale invasive phenotypes**

Laser confocal micrographs of maximum intensity projected images of rBM-coated cell clusters embedded in Type 1 collagen. (A) Immortalized mammary epithelial cells (HMLE), form growth-arrested acinar-like structures. Merge inset shows a single acinar-like structure with a lumen (B) Non-invasive breast cancer cells (MCF7) show a carcinoma-in-situ-like phenotype. Merge inset shows a cell-filled MCF7 cluster. (C) Invasive breast cancer cells (MDA-MB-231) show multiscale invasion. Scale bar = 100 μm.
