# Supplementary material for: An Interplay Between Reaction-Diffusion and Cell-Matrix Adhesion Regulates Multiscale Invasion in Early Breast Carcinomatosis
Source: Front Physiol. 2019 Aug 13;10:790. doi: 10.3389/fphys.2019.00790 (PMC6700745; doi:10.3389/fphys.2019.00790)
Supplement: Supplementary file 3 [file Data_Sheet_2.docx]

**Exploration of the relaxation criterion of Activator-Inhibitor diffusivity ratio:**

Rigorous work on cooperativity in activator-inhibitor reaction dynamics by Diambra and colleagues shows a possible relaxation in the threshold of activator-inhibitor diffusivity ratio for spatial symmetry-breaking (Diambra et al., 2015). Motivated by this work, we explored the possibility of multiscale invasion under relaxed activator-inhibitor diffusivity ratios. An essential parameter, which can intuitively be hypothesized to regulate invasion under altered relative diffusion of MMP/TIMP is the [MMP]/[TIMP] at a given location that degrades matrix at that location. For our experiments we assumed this to be 2. However, plotting the relation between the D_a_/D_i_ and the threshold max[A]/[I] results in the parameter space being broken up into two sub-spaces. The parametric combinations that lie above the line disallow for multiscale invasion. On the other hand, those below are permissive for multiscale invasion. This suggests that multiscale invasion can occur under relaxed Da/Di values if the Inhibitor’s effect at attenuating the activator’s function (degradation) weakens.


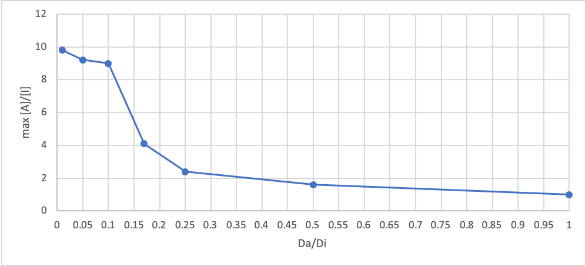


No multiscale invasion

multiscale invasion

**Quantitative comparison between in culture- and in silico multiscale invasion:**

In order to quantitatively compare the observation of multiscale invasion in culture and in our computational model, we have first calculated the initial size of the cancer cell cluster (C1) before beginning the assay/simulation, the size of the cluster at a given time point/MCS (C2) and the total area enclosing the invading single cells and the continuous cell cluster (C3) in culture and in silico. We then define for each given time point (or MCS) two metrics: the first measures bulk/collective cell invasion and is given by (C2-C1/C1). The second, which gives a sense of single cell invasion is given by (C3-C2)/C1.


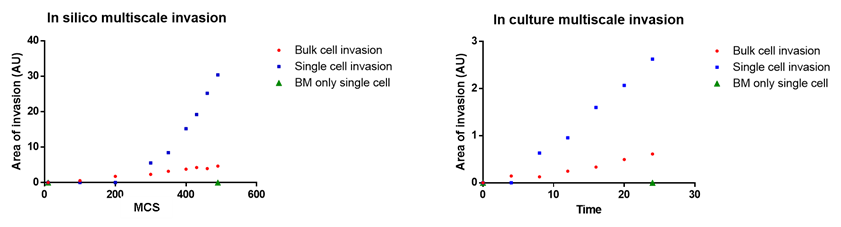


Upon plotting both these parameters along axes of time/MCS, we observe that the metric for single cell invasion (blue) trails behind that for bulk invasion (red) at very early time steps and then abruptly increases towards the end of the assay (or simulation). On the other hand, the metric for bulk or collective invasion shows a slow temporal rise. There is no singular cell invasion to be seen for assays in which the presence of fibrillar matrix was avoided (or not modeled; green).
