# Supplementary material for: An Interplay Between Reaction-Diffusion and Cell-Matrix Adhesion Regulates Multiscale Invasion in Early Breast Carcinomatosis
Source: Front Physiol. 2019 Aug 13;10:790. doi: 10.3389/fphys.2019.00790 (PMC6700745; doi:10.3389/fphys.2019.00790)
Supplement: Supplementary file 4 [file Data_Sheet_3.docx]

MCS and time conversion:

There are several ways by which the conversion between MCS and actual time of experiments can be computed. These include:

1. Population doubling time comparison
2. Diffusion and/or secretion rates comparison (which we have performed)
3. Comparison of cell speed in active motility (such as has been employed in Kumar et al Scientific Reports, 2016, which we have not implemented since we did not incorporate active motility in our model)

The prime objective of our current model is to explore conditions for the permissibility of the multiscale invasion phenotype as well as to explore whether the same model can explain a panoply of phenotypes obtained under experimental perturbations. In order to derive a relationship between MCS and time for our model, we would choose diffusion rate from reaction diffusion module as a reference, as suggested by the first reviewer.

Calculation:

Diffusion coefficient of MMP from literature: 1.0 x 10^-9^ cm^2^.s^-1^

Ref: Kumar et al, Sci Rep, 2016

Diffusion rate of A in our model: 0.01 pixel^2^.MCS^-1^

Approximated maximum length scale of the elongated MDA-MB-231 cells = 40 µm

In-silico “CELL”: 4 pixel * 4 pixel square initial cell size

So, correlating these length scales we get,

4 pixel = 40 µm; or, 1 mm = 100 pixel ; or, 1 cm = 10^3^ pixel

Now, by comparing the diffusion rates of MMP from literature and of A from our model we get,

$$\frac{1.0 \times{10}^{-9} \times\left( {10}^{3} \right)^{2} pixel unit}{second}=\frac{0.01 pixel unit}{MCS}$$

[1 cm^2^ = $\left( {10}^{3} \right)^{2} pixel unit$]

$$=>MCS=10 seconds$$

This value is not realistic if comparison is done with the population doubling time. To solve that issue the growth rate of the ‘CELL’ needs to be optimized. Such optimization can also be performed with changing diffusion and secretion rates. The following graph shows that dividing the rates (all secretion and diffusion rates except growth rate) in the model increases the MCS required for reaching certain invasion area with same initial cell mass.

Here,

Y axis, MCS’’ => MCS at which ‘area of optimum enclosing circle’ reaches 50000 units.

X axis, divisor => factor by which all the rates (except growth rate) were divided.

However, even with MCS = 10 sec, multiscale invasion is observed by about 600-700 MCS steps *in silico*. In consonance with the *in silico* time scale, multiscale invasion is discernible in experimental cultures within 2-3 hours.
