# Supplementary figures and images for: An Interplay Between Reaction-Diffusion and Cell-Matrix Adhesion Regulates Multiscale Invasion in Early Breast Carcinomatosis
Source: Front Physiol. 2019 Aug 13;10:790. doi: 10.3389/fphys.2019.00790 (PMC6700745; doi:10.3389/fphys.2019.00790)

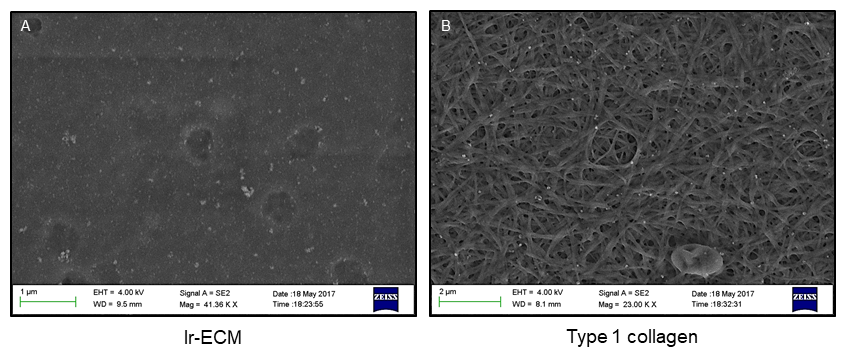

Supplement: Supplementary file 5 [file Image_1.TIF]

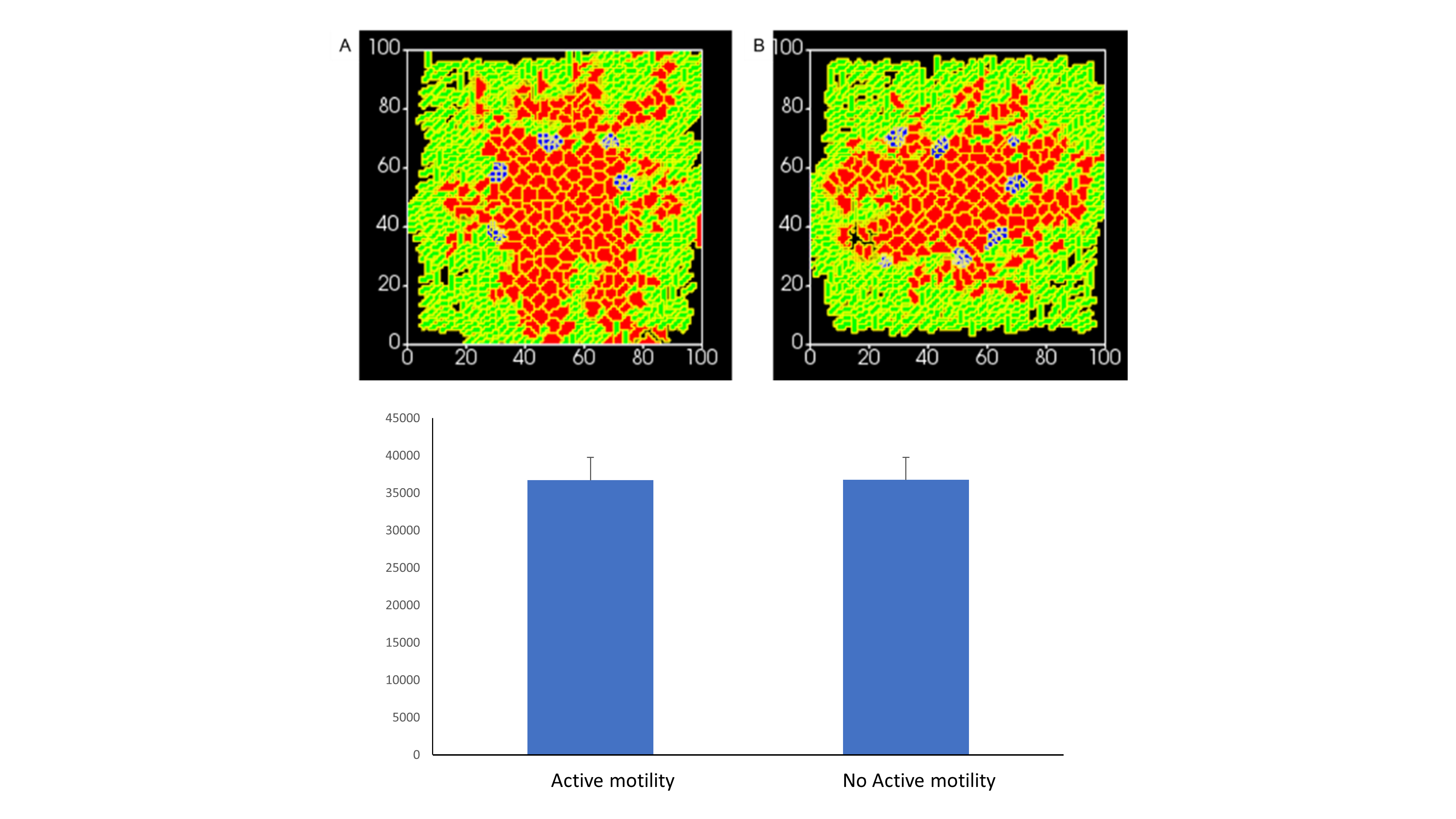

Supplement: Supplementary file 6 [file Image_2.TIF]

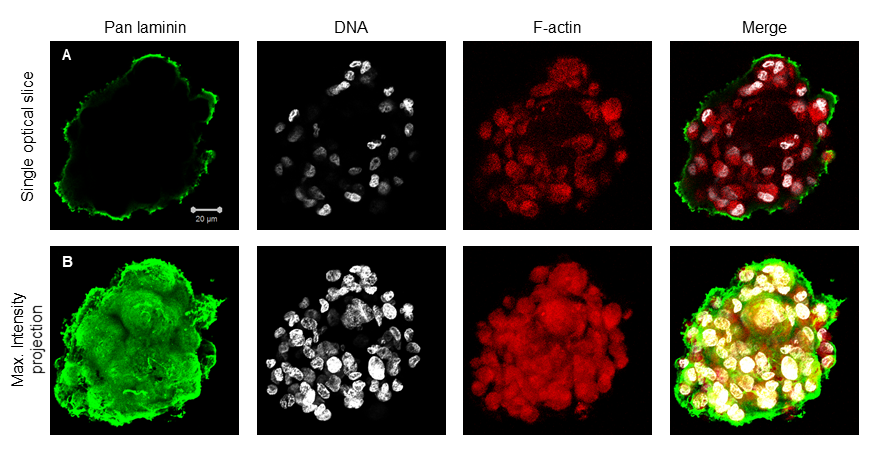

Supplement: Supplementary file 7 [file Image_3.TIF]

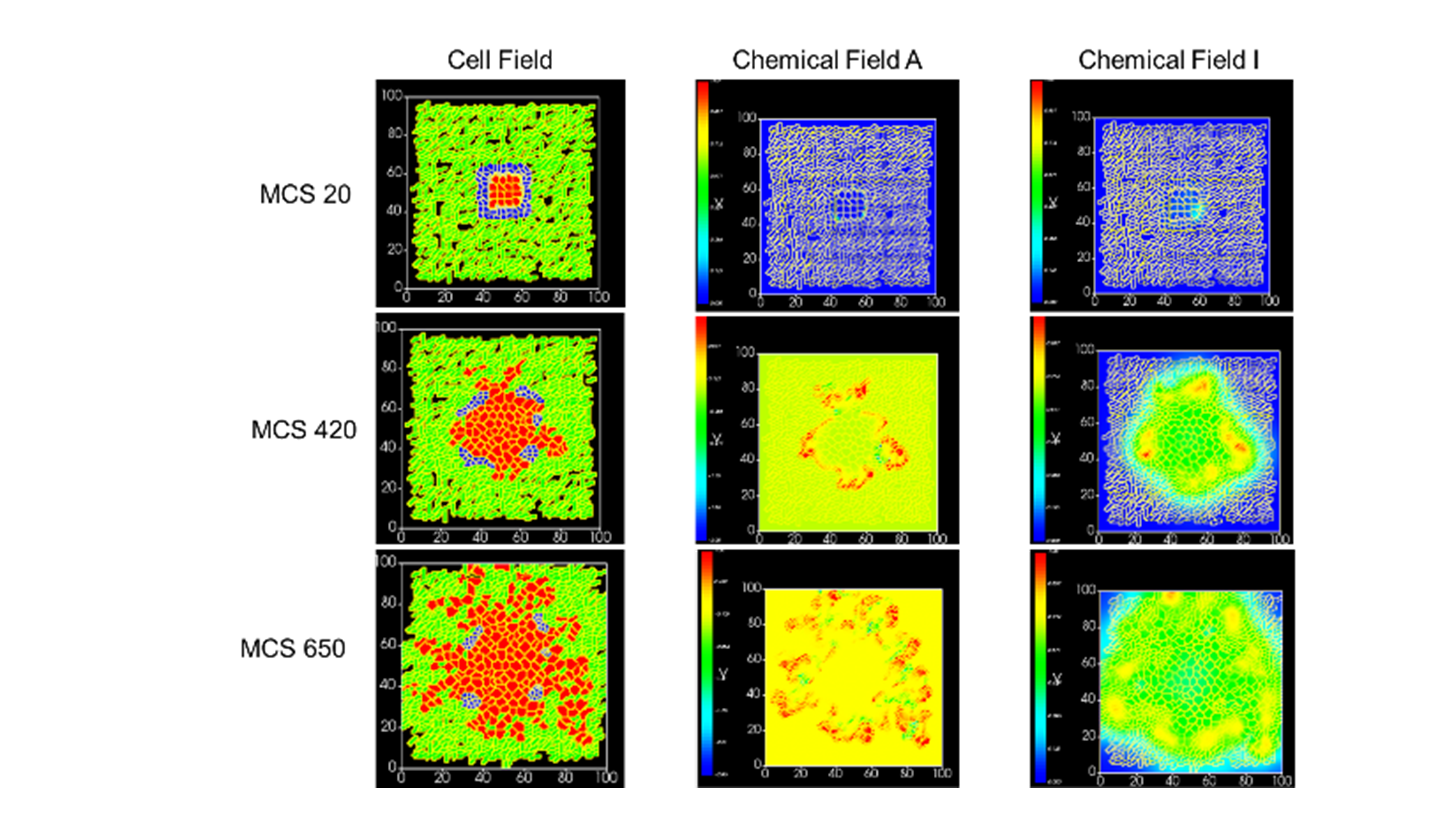

Supplement: Supplementary file 8 [file Image_4.TIF]

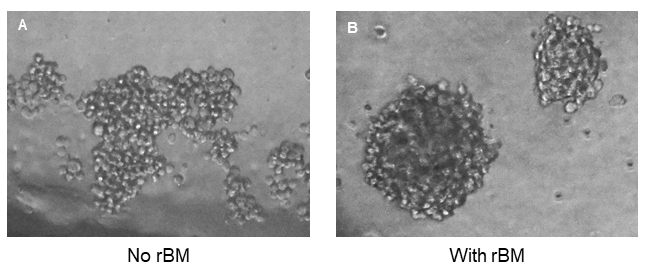

Supplement: Supplementary file 9 [file Image_5.TIF]

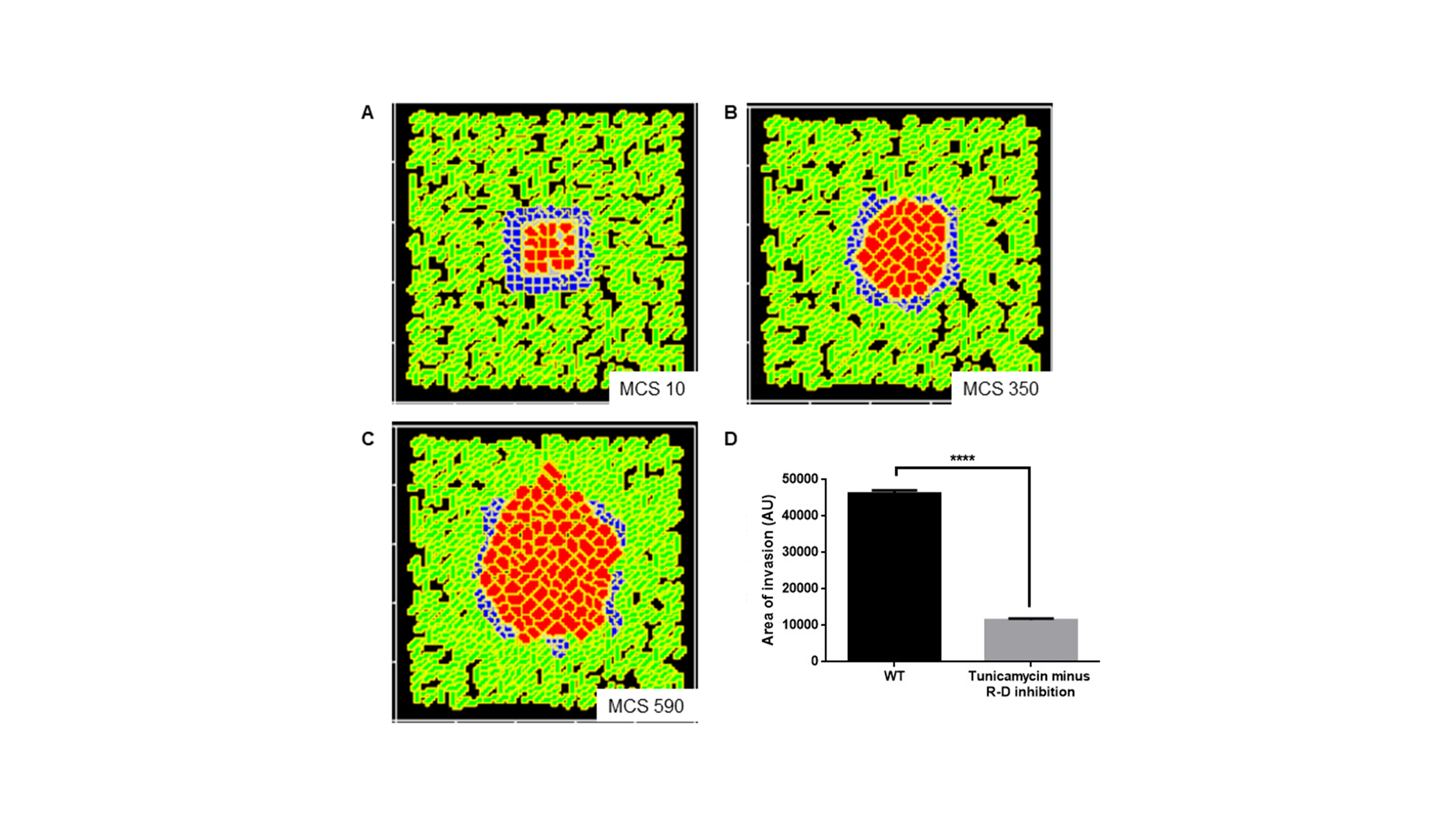

Supplement: Supplementary file 10 [file Image_6.tif]

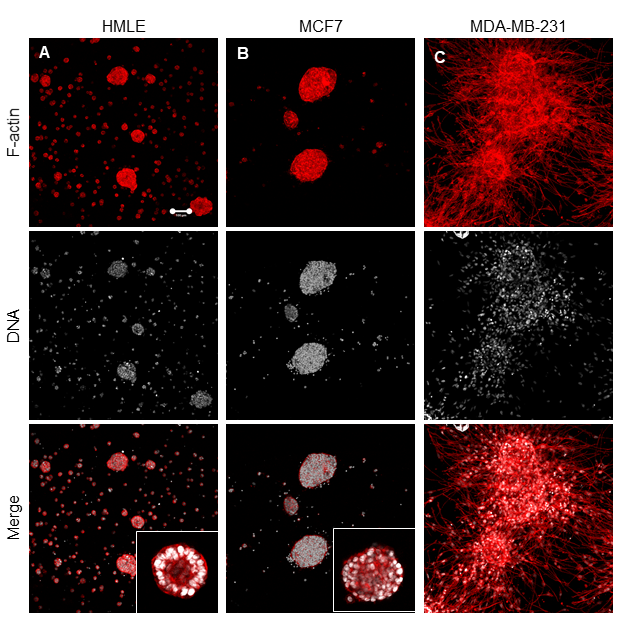

Supplement: Supplementary file 11 [file Image_7.TIF]
